# Supplementary figures and images for: Integrins regulate hERG1 dynamics by girdin-dependent Gαi3: signaling and modeling in cancer cells
Source: Life Sci Alliance. 2023 Nov 3;7(1):e202302135. doi: 10.26508/lsa.202302135 (PMC10624597; doi:10.26508/lsa.202302135)

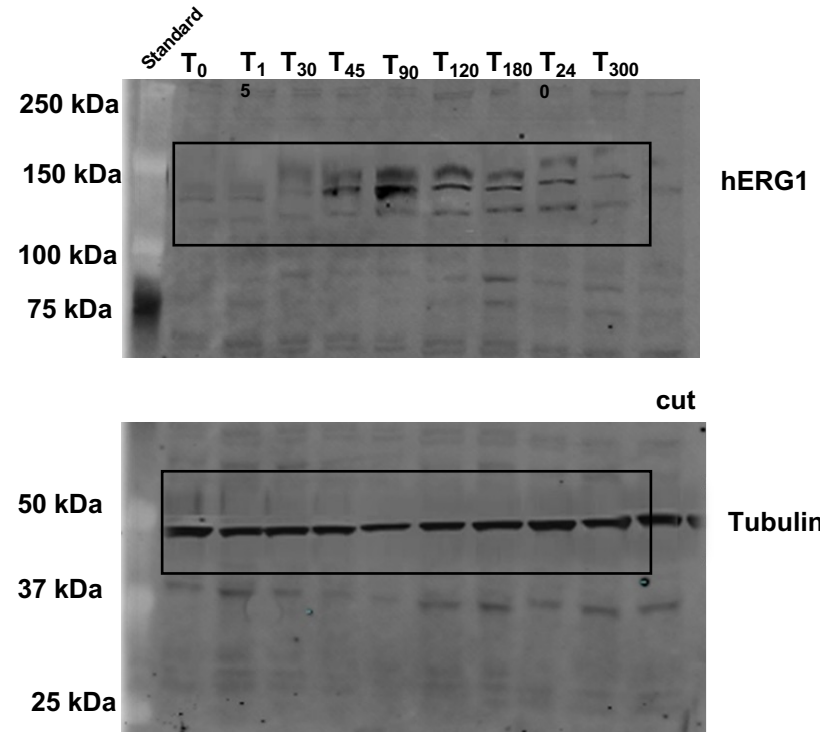

SourceDataForFigure2B

Supplement: Supplementary file 1 [file LSA-2023-02135_SdataF2.pdf]

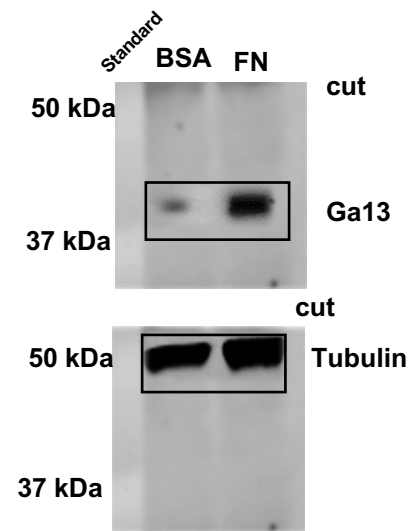

SourceDataForFigure3D

Supplement: Supplementary file 2 [file LSA-2023-02135_SdataF3.1.pdf]

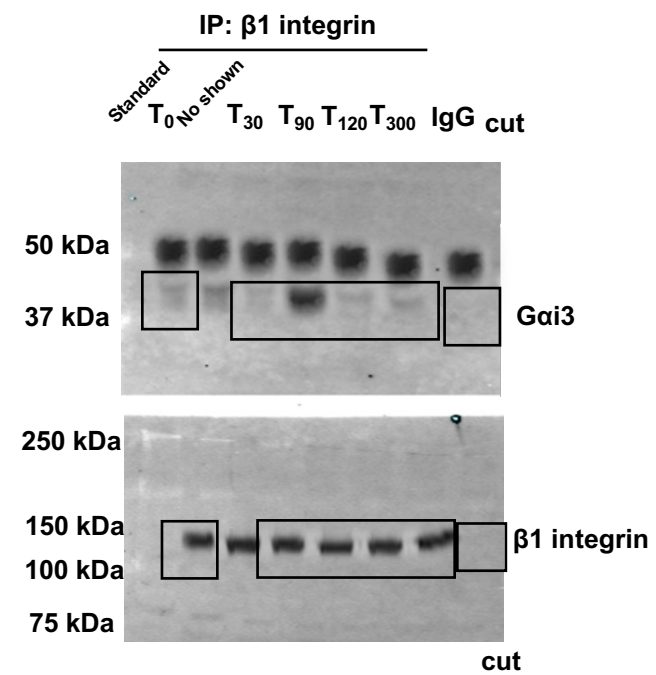

SourceDataForFigure3E

Supplement: Supplementary file 3 [file LSA-2023-02135_SdataF3.2.pdf]

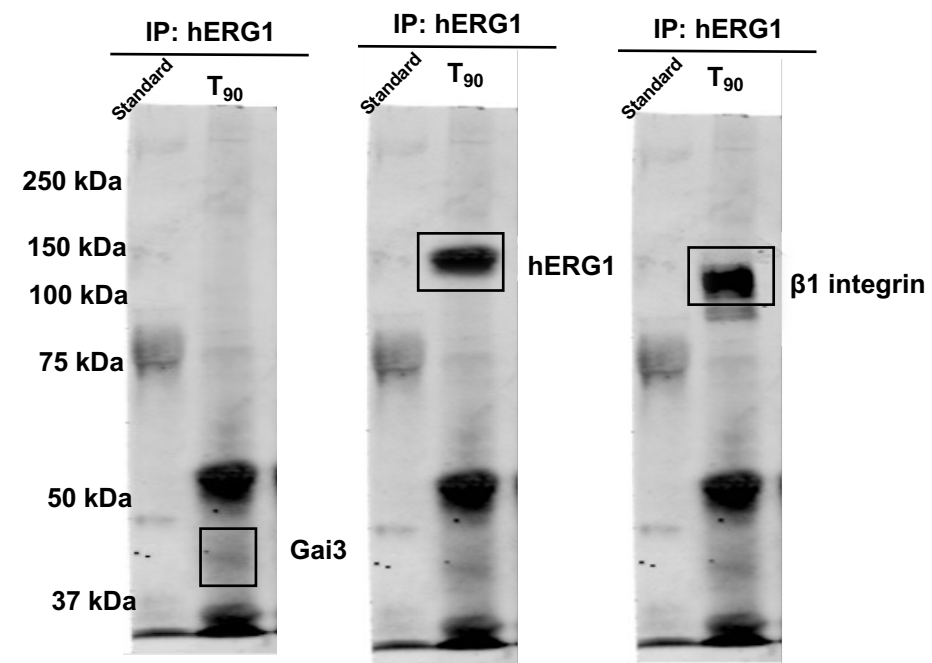

SourceDataForFigure3F

Supplement: Supplementary file 4 [file LSA-2023-02135_SdataF3.3.pdf]

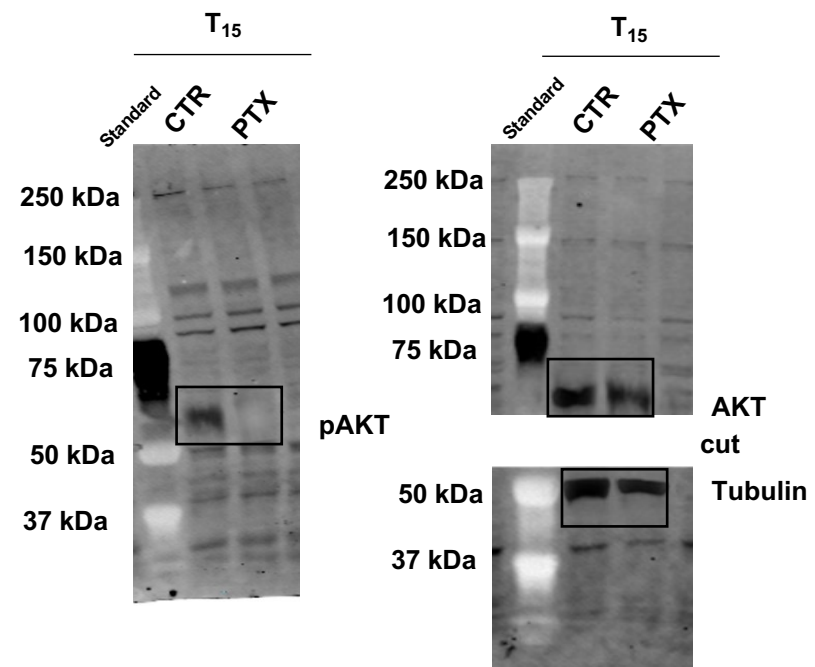

SourceDataForFigure3G

Supplement: Supplementary file 5 [file LSA-2023-02135_SdataF3.4.pdf]

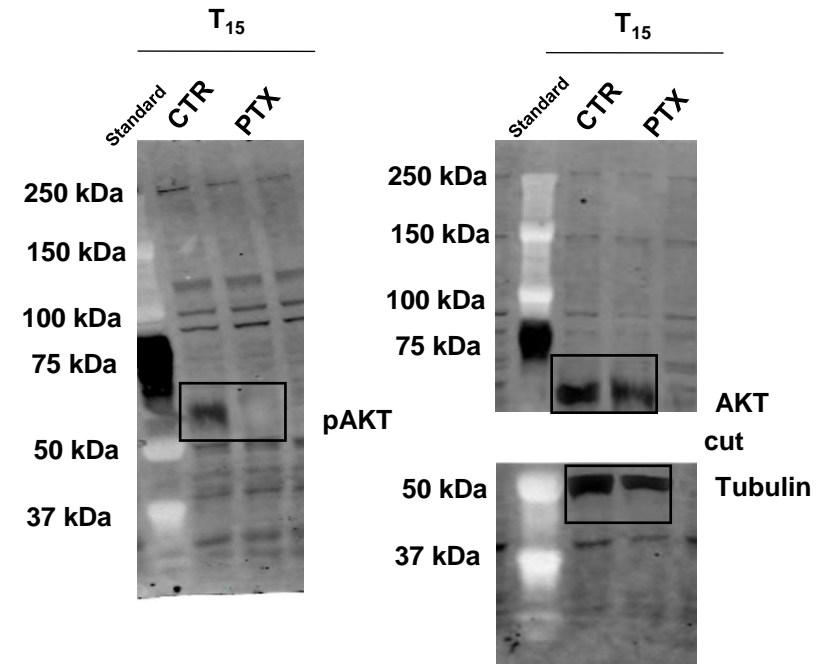

SourceDataForFigure3L

Supplement: Supplementary file 6 [file LSA-2023-02135_SdataF3.5.pdf]

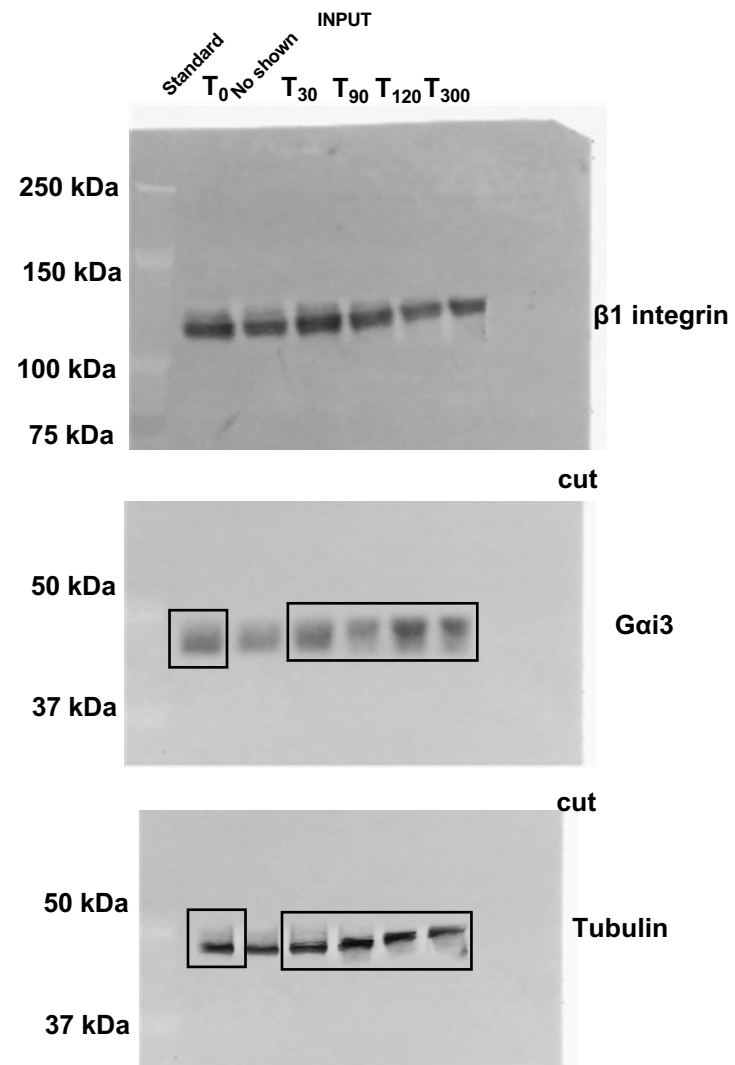

SourceDataForSupplementaryFigureS3A

Supplement: Supplementary file 7 [file LSA-2023-02135_SdataFS3.1.pdf]

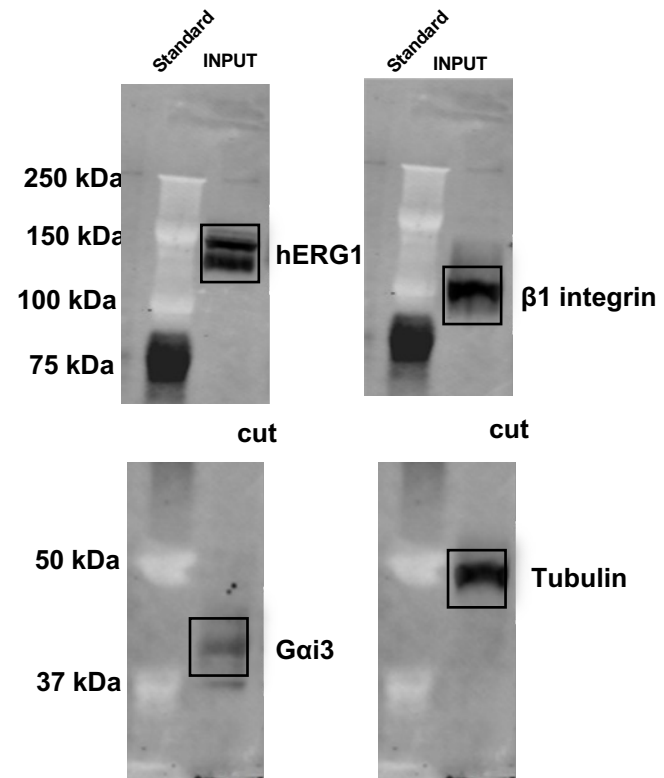

SourceDataForSupplementaryFigureS3C

Supplement: Supplementary file 8 [file LSA-2023-02135_SdataFS3.2.pdf]

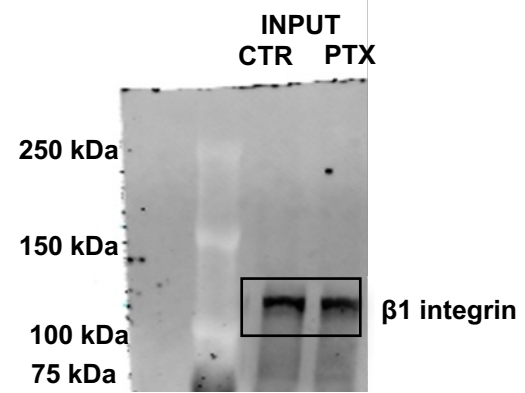

cut

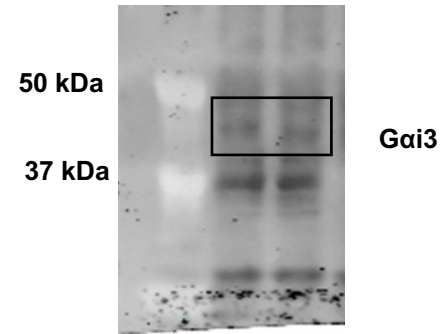

cut

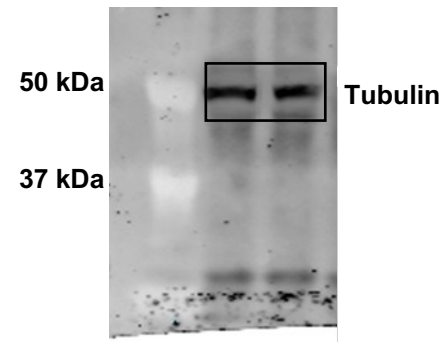

SourceDataForSupplementaryFigureS3D

Supplement: Supplementary file 9 [file LSA-2023-02135_SdataFS3.3.pdf]

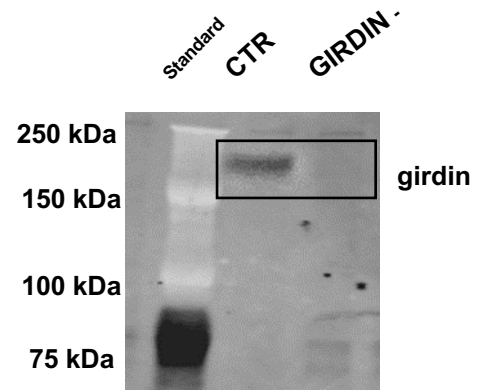

cut

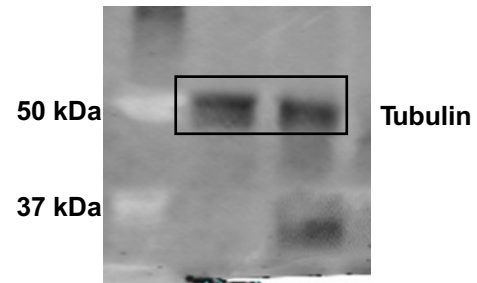

SourceDataForSupplementaryFigureS3F

Supplement: Supplementary file 10 [file LSA-2023-02135_SdataFS3.4.pdf]

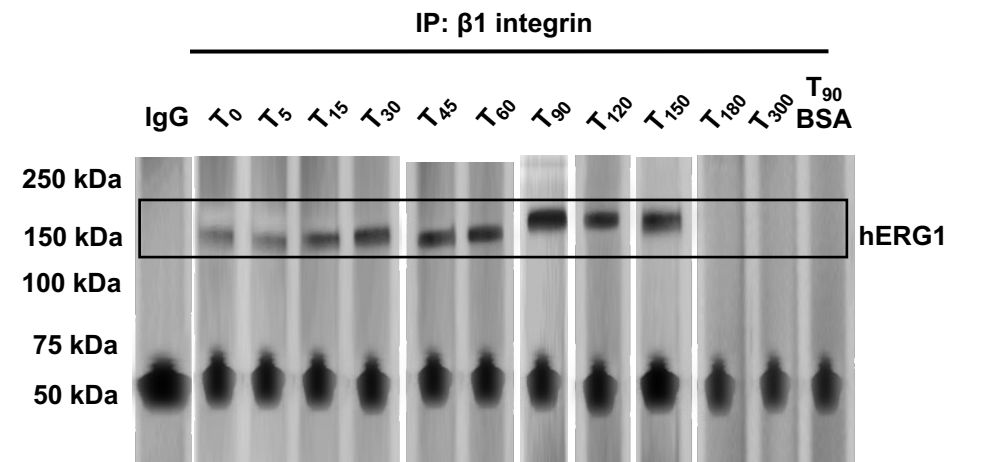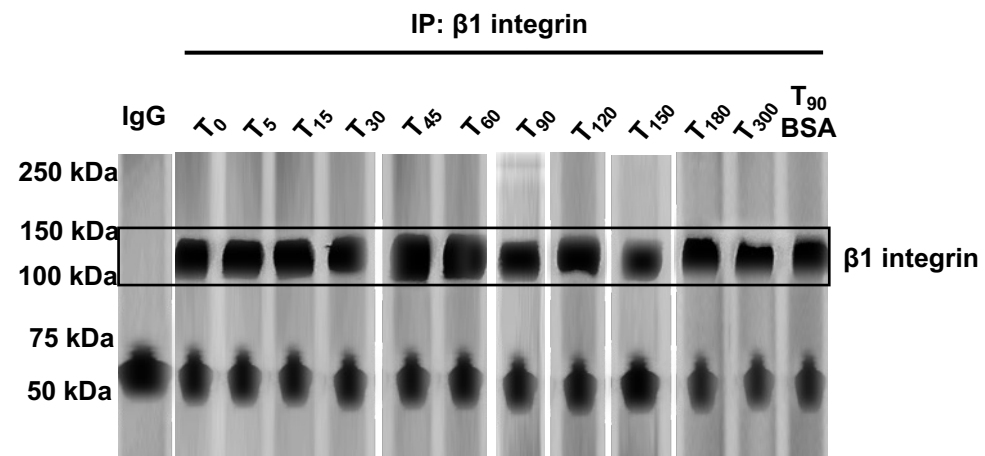

SourceDataForFigure4

Supplement: Supplementary file 11 [file LSA-2023-02135_SdataF4.pdf]

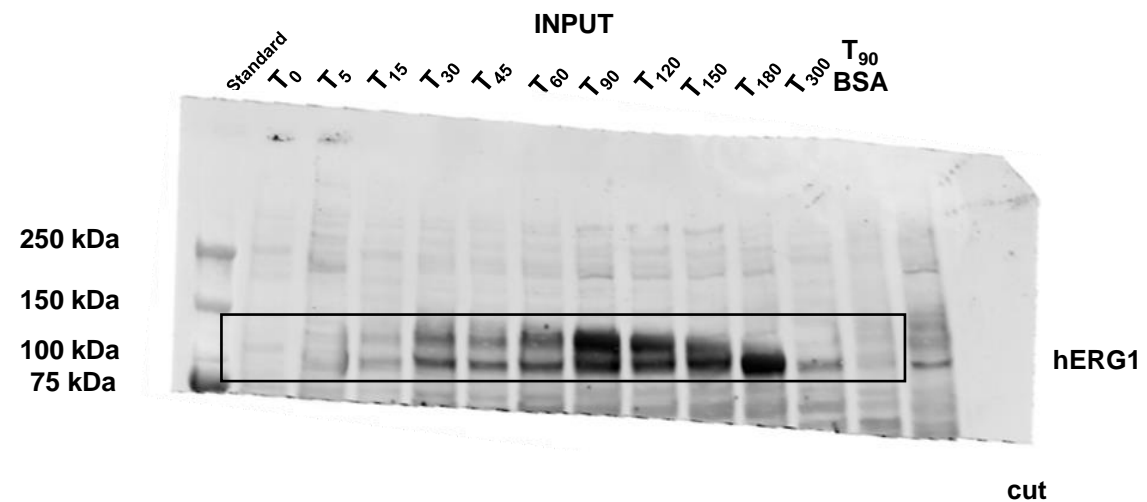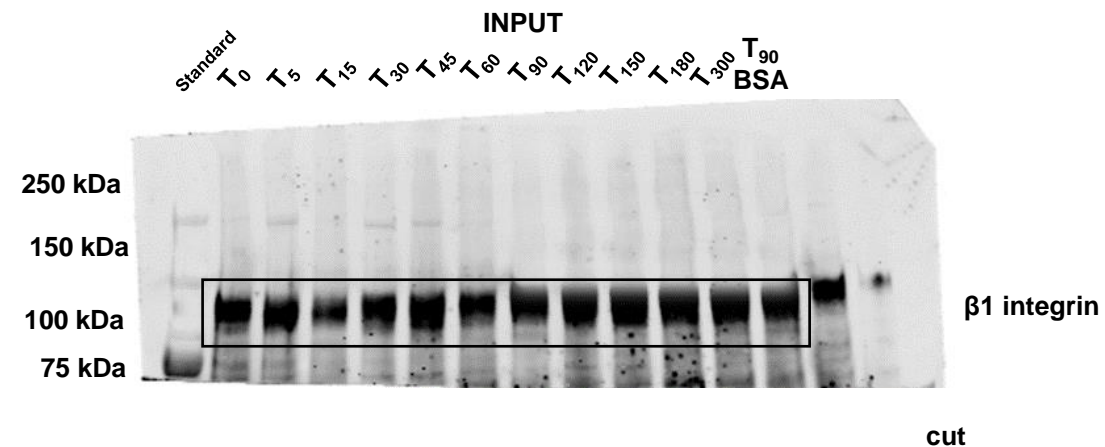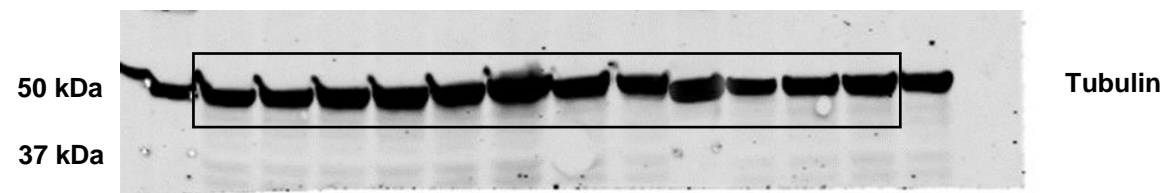

SourceDataForSupplementaryFigureS4A

Supplement: Supplementary file 12 [file LSA-2023-02135_SdataFS4.pdf]

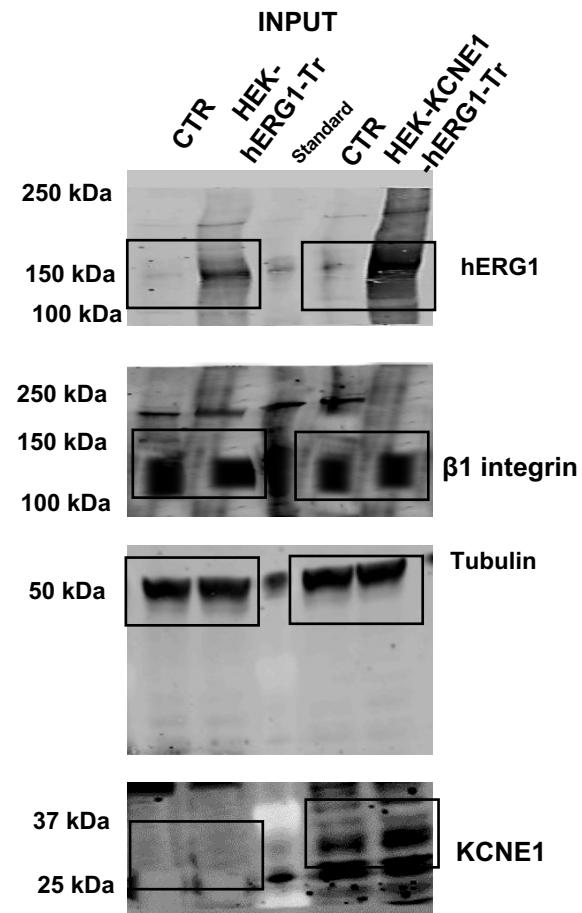

SourceDataForSupplementaryFigureS5B

Supplement: Supplementary file 14 [file LSA-2023-02135_SdataFS5.2.pdf]

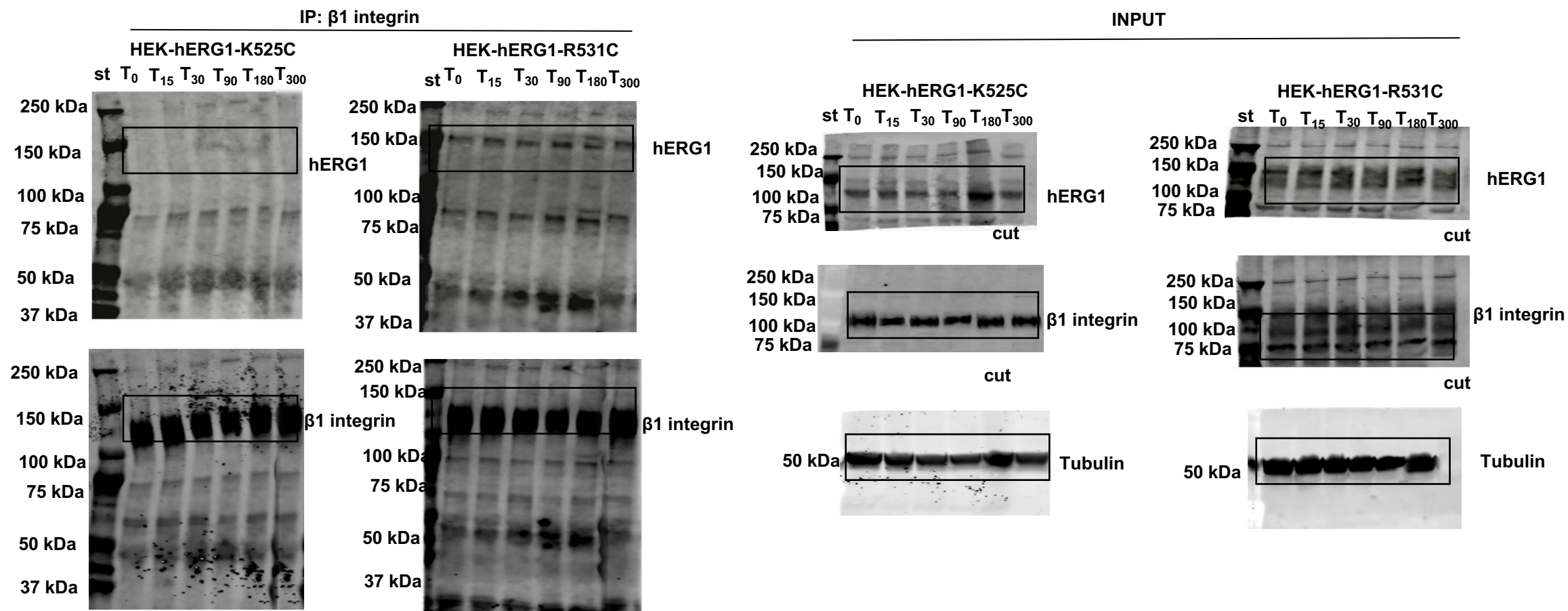

SourceDataForSupplementaryFigureS5D

Supplement: Supplementary file 15 [file LSA-2023-02135_SdataFS5.3.pdf]

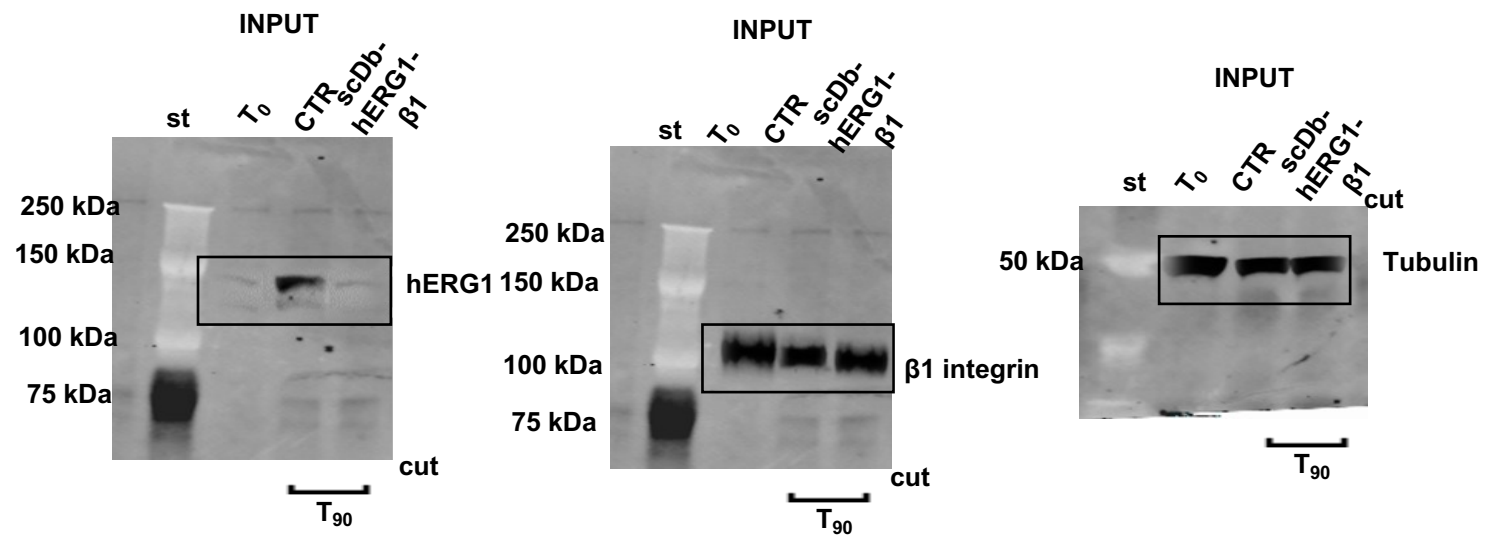

SourceDataForSupplementaryFigureS5F

Supplement: Supplementary file 16 [file LSA-2023-02135_SdataFS5.4.pdf]

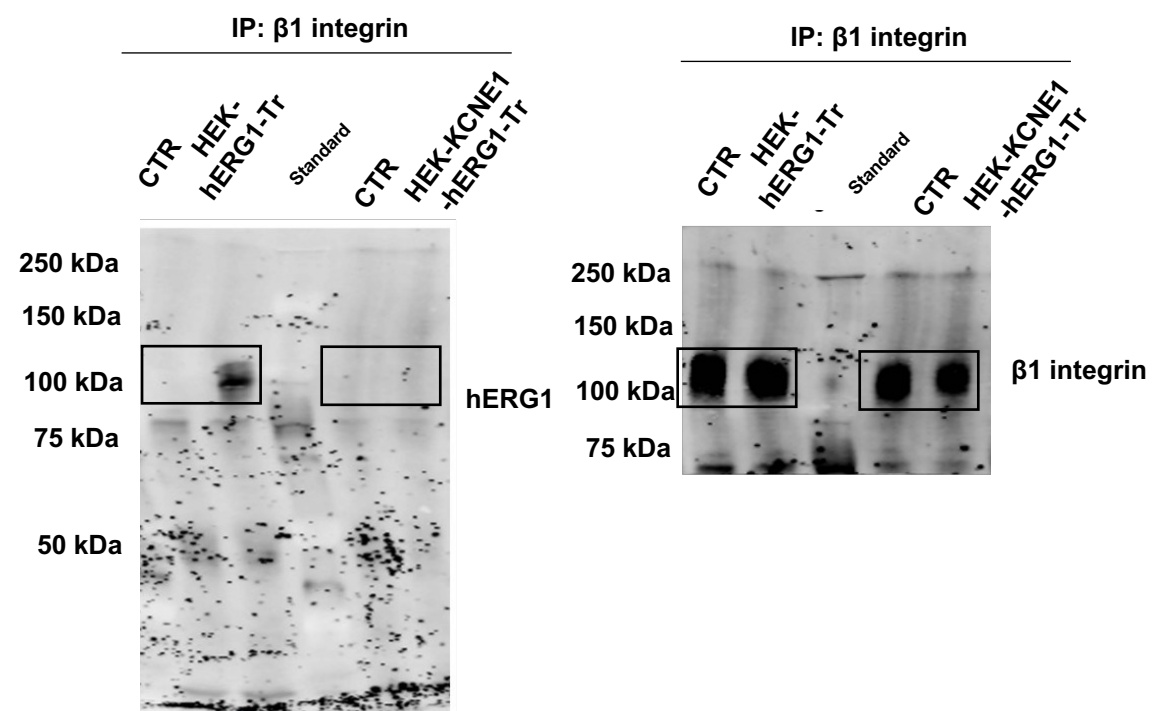

SourceDataForFigure5C

Supplement: Supplementary file 17 [file LSA-2023-02135_SdataF5.1.pdf]

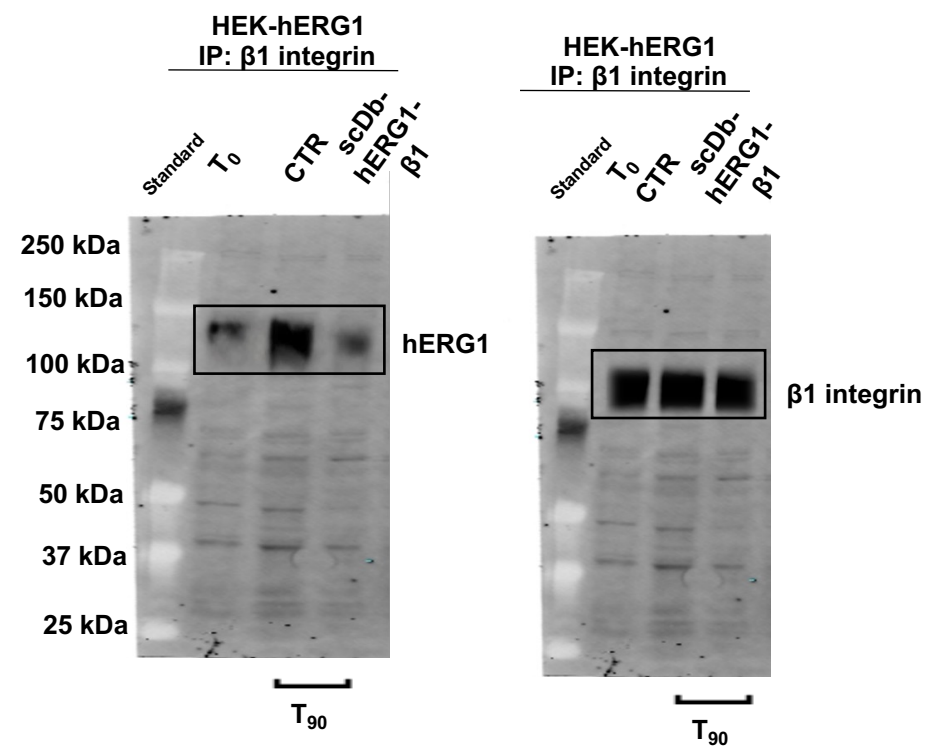

SourceDataForFigure5J

Supplement: Supplementary file 18 [file LSA-2023-02135_SdataF5.2.pdf]

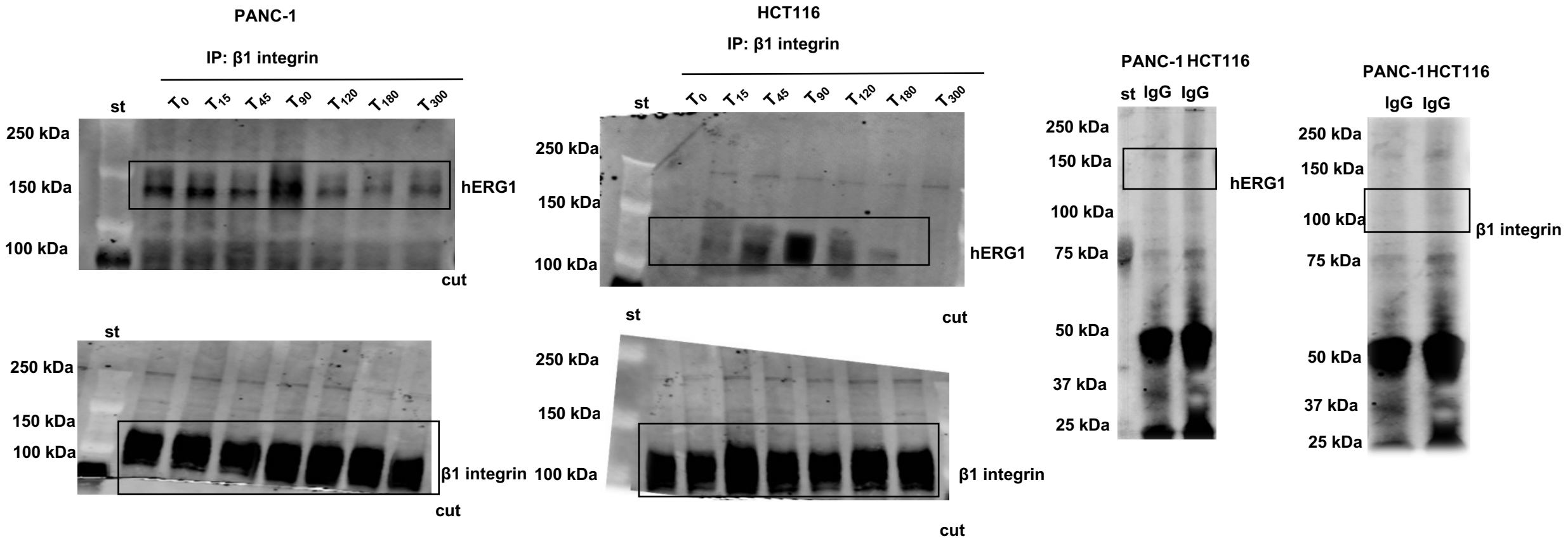

SourceDataForFigure6E

Supplement: Supplementary file 20 [file LSA-2023-02135_SdataF6.pdf]

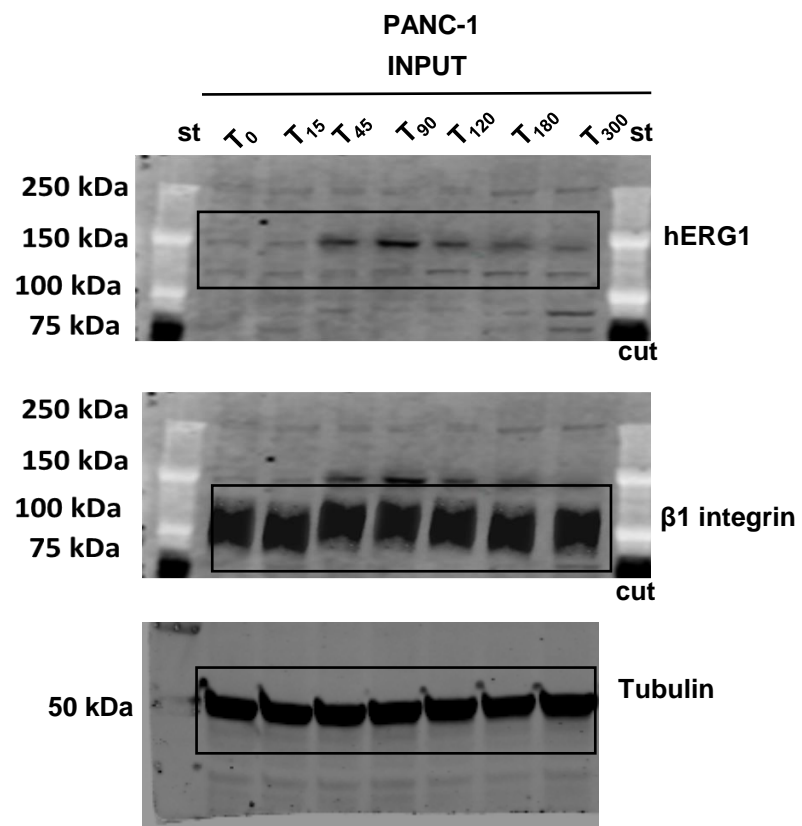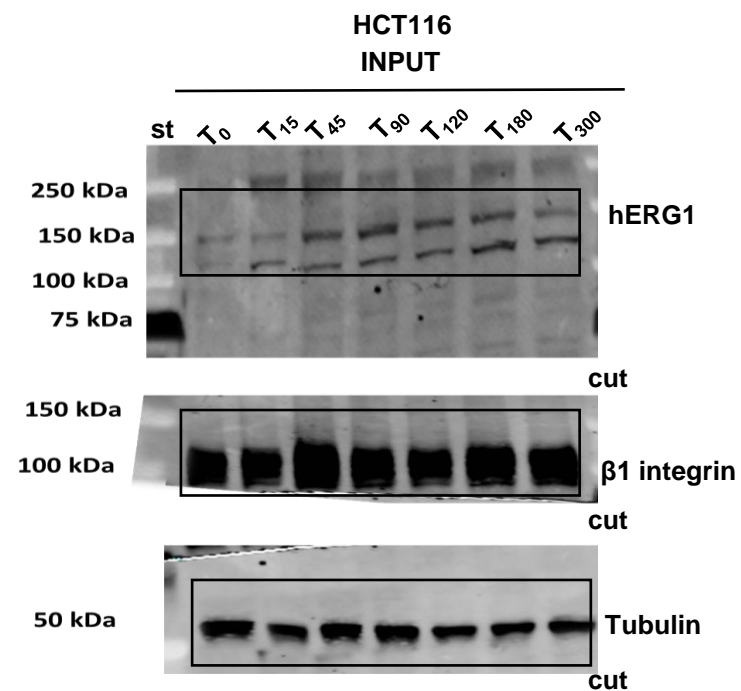

SourceDataForSupplementaryFigureS6C

Supplement: Supplementary file 21 [file LSA-2023-02135_SdataFS6.pdf]

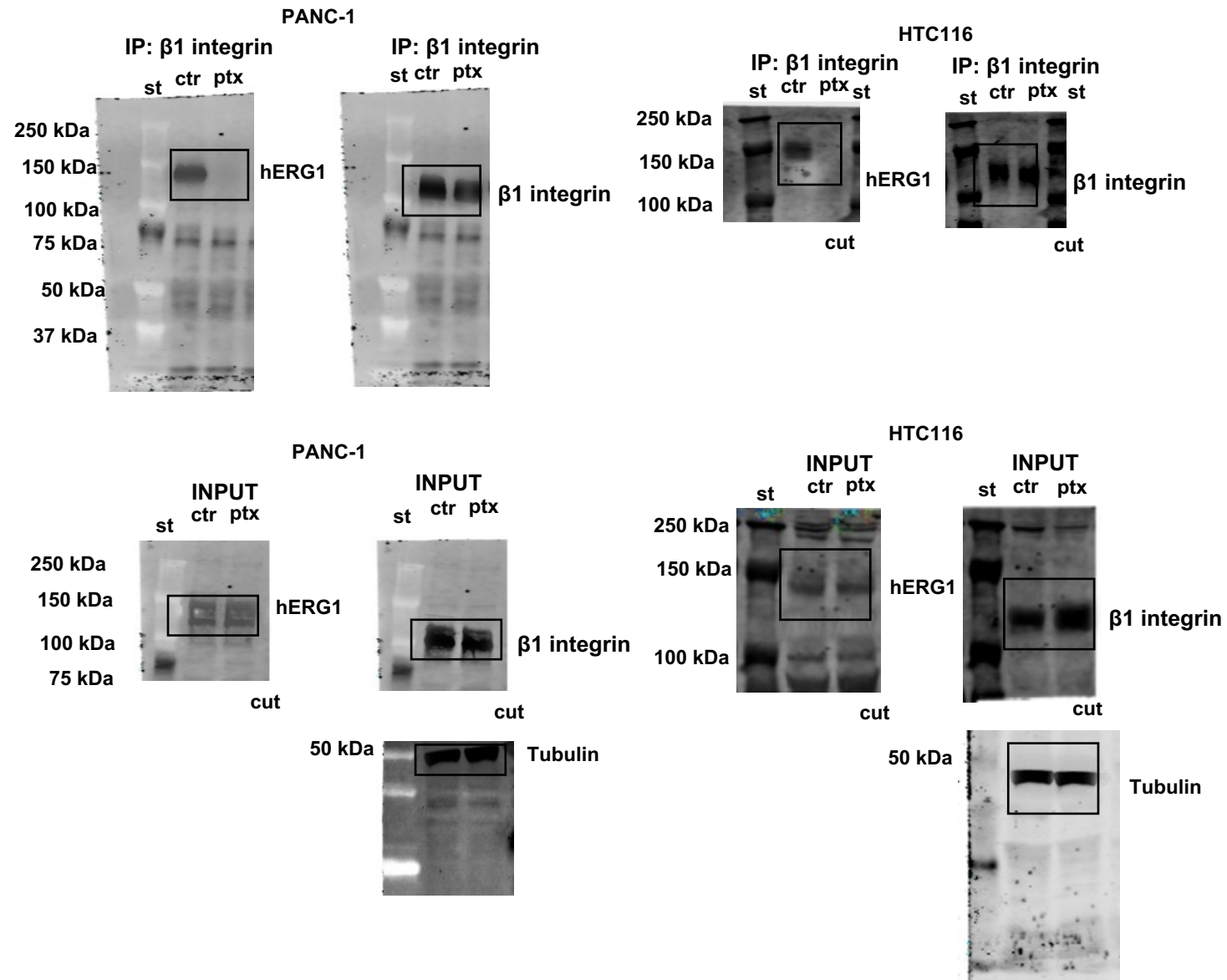

SourceDataForSupplementaryFigureS7G

Supplement: Supplementary file 22 [file LSA-2023-02135_SdataFS7.1.pdf]

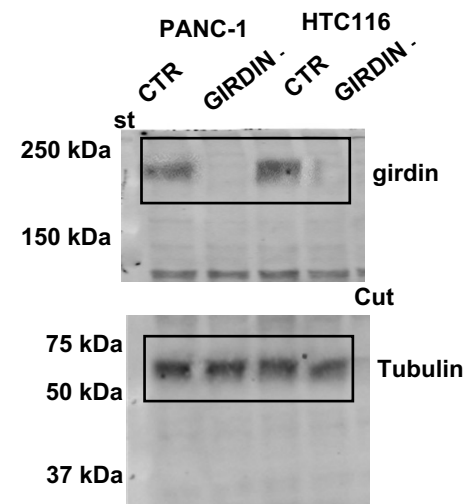

SourceDataForSupplementaryFigureS7H

Supplement: Supplementary file 23 [file LSA-2023-02135_SdataFS7.2.pdf]

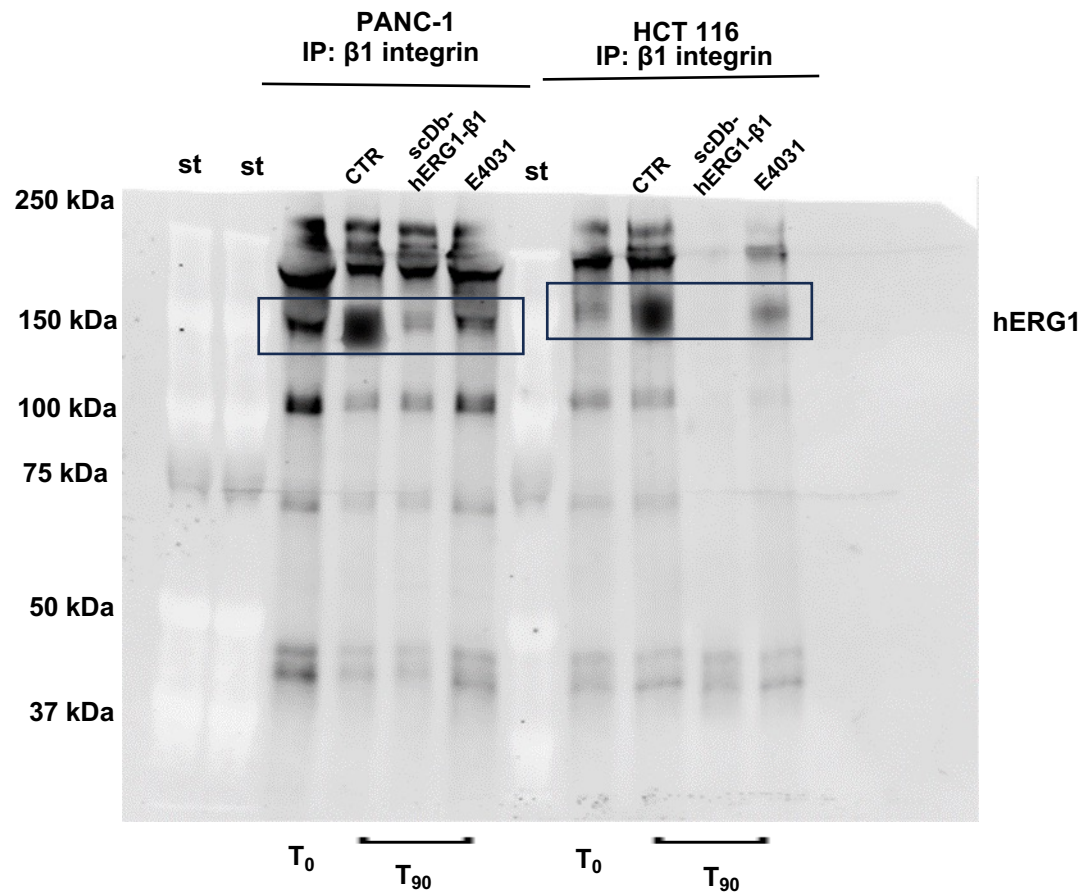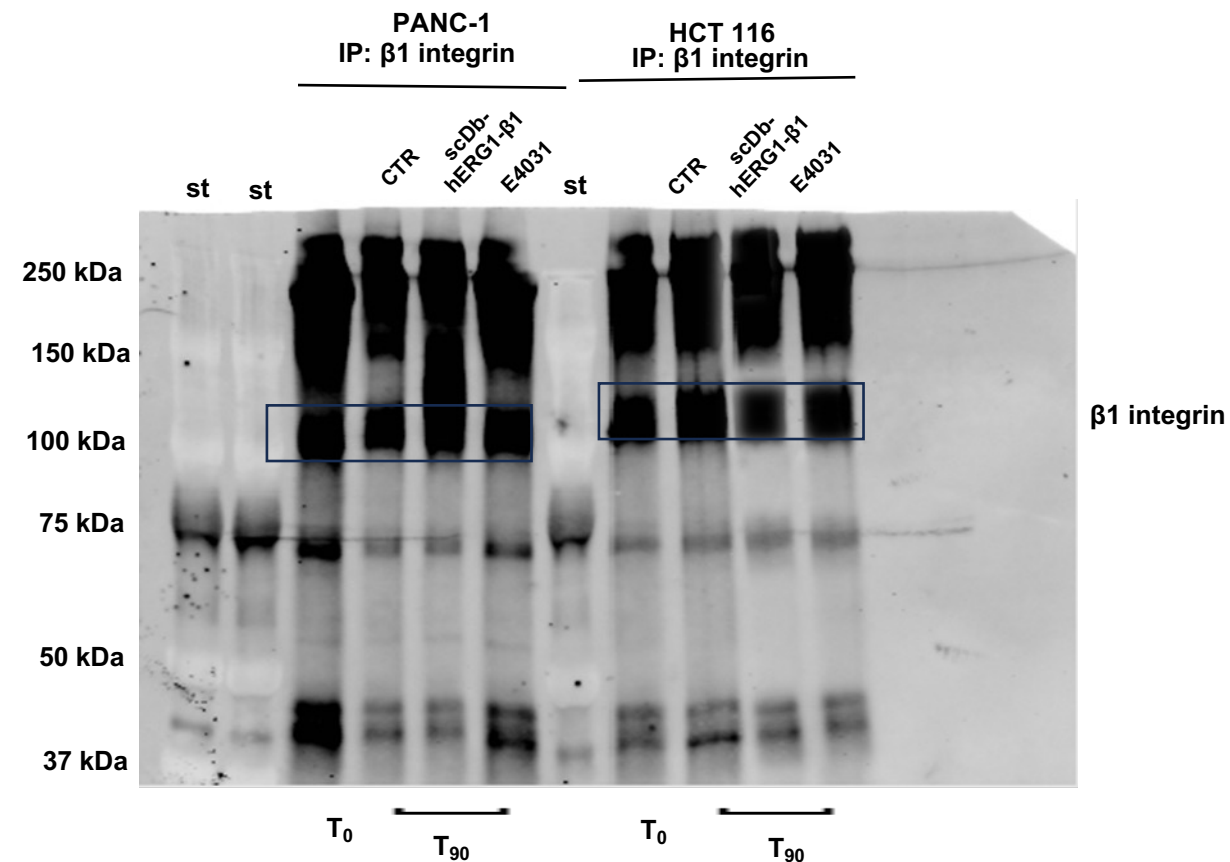

SourceDataForFigure8B

Supplement: Supplementary file 24 [file LSA-2023-02135_SdataF8.pdf]

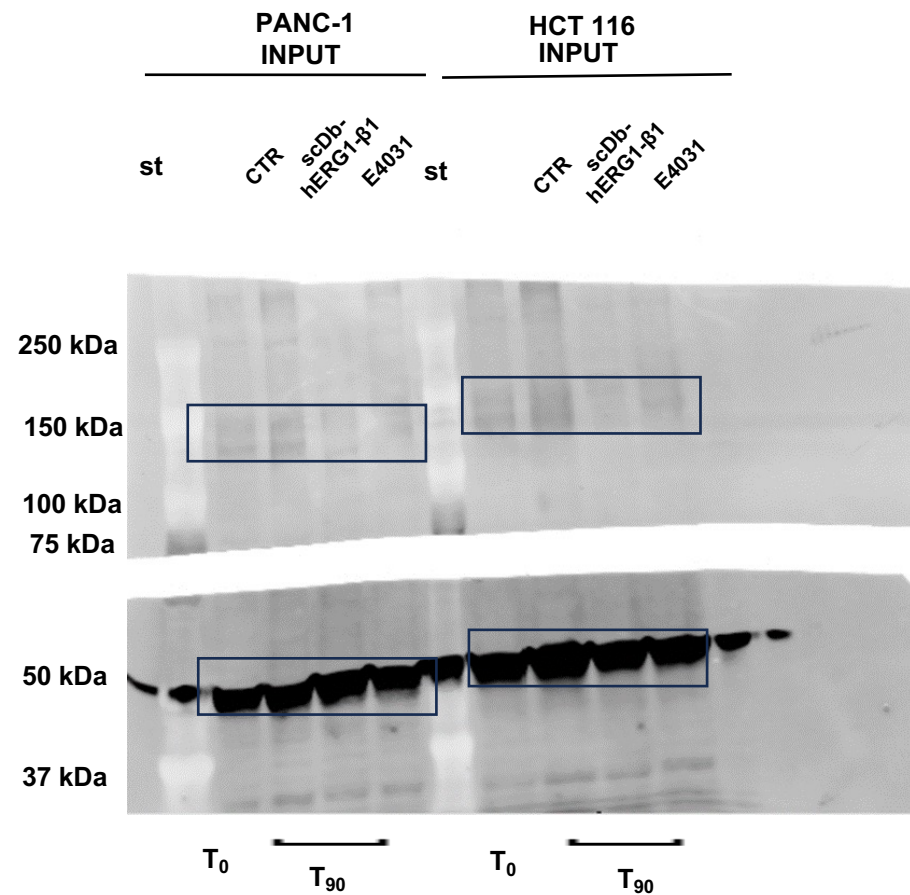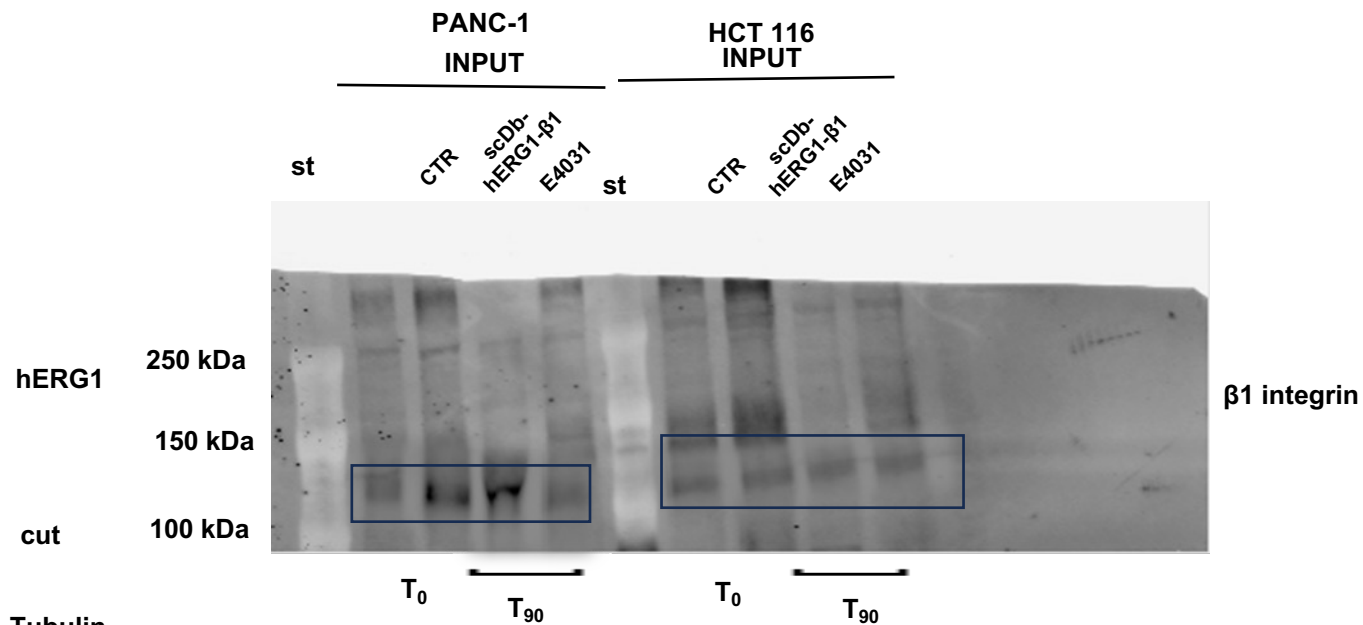

SourceDataForSupplementaryFigureS8A

Supplement: Supplementary file 25 [file LSA-2023-02135_SdataFS8.pdf]

HEK-hERG1

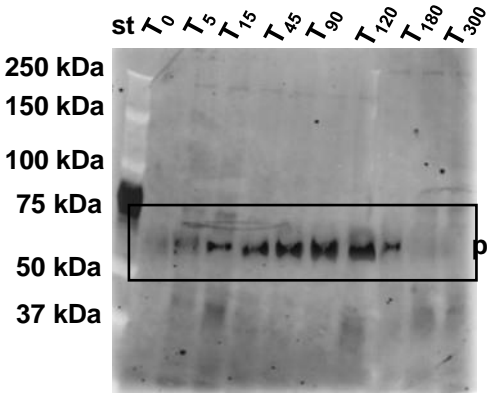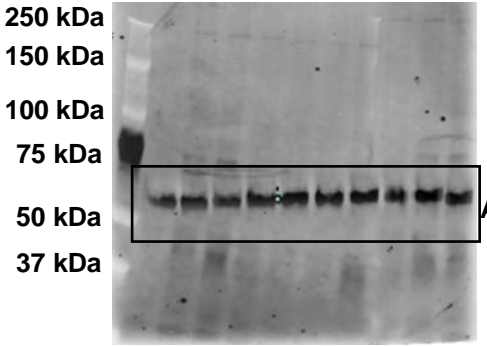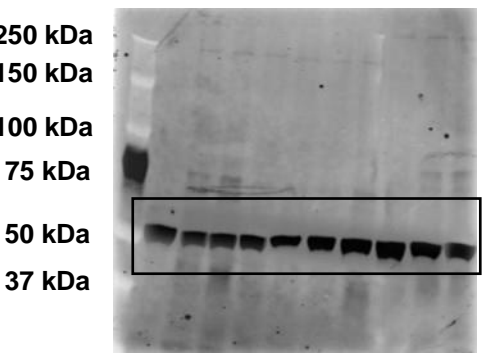

PANC-1

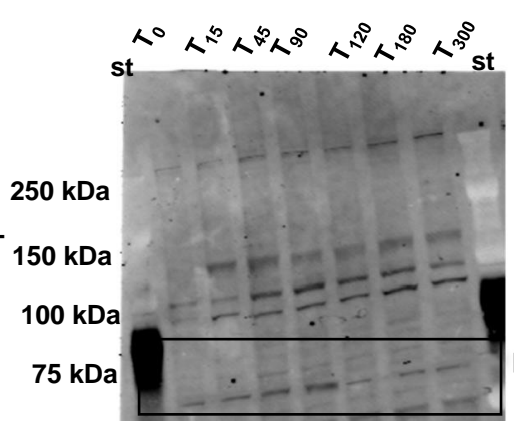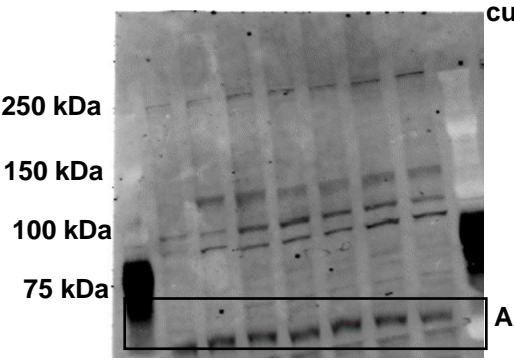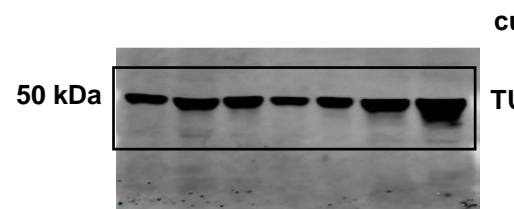

HCT116

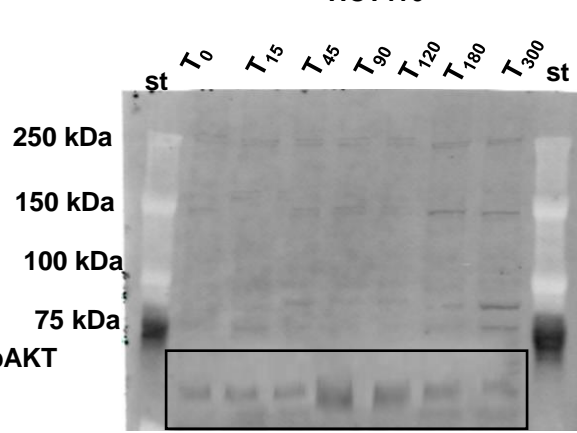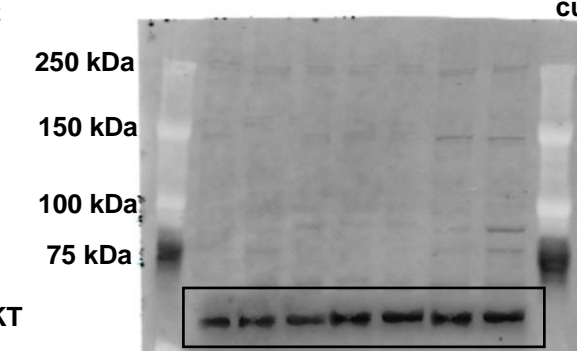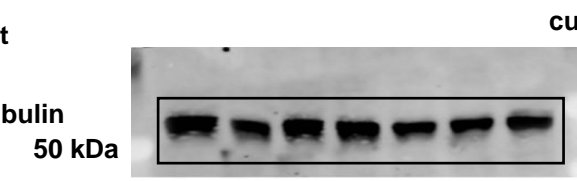

HEK-hERG1

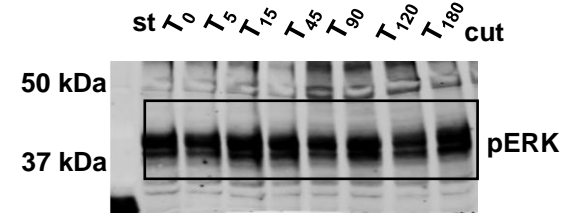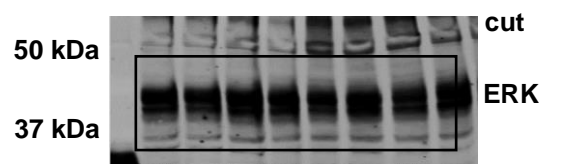

PANC-1

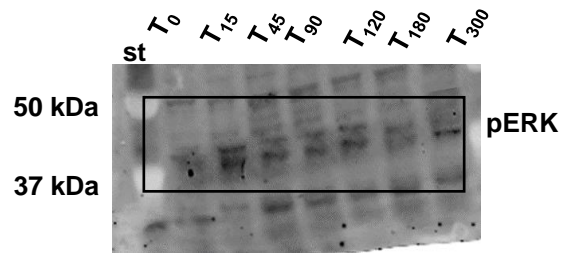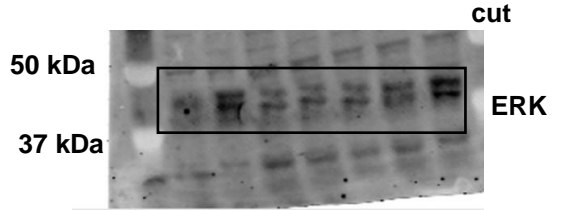

HCT116

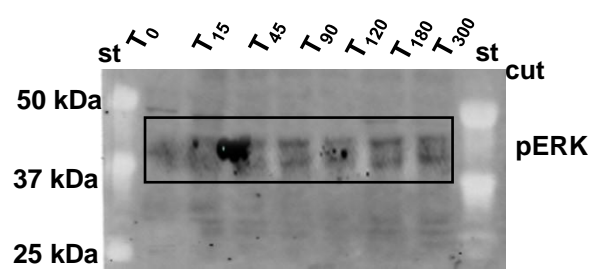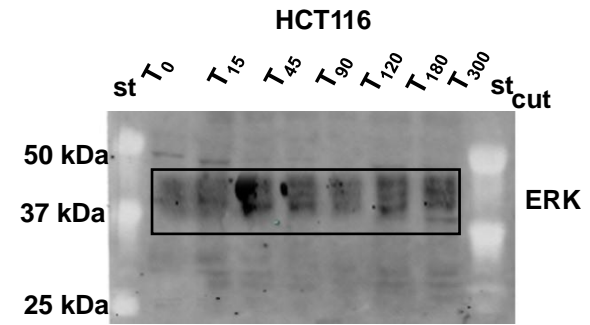

Supplement: Supplementary file 26 [file LSA-2023-02135_SdataFS9.1.pdf]

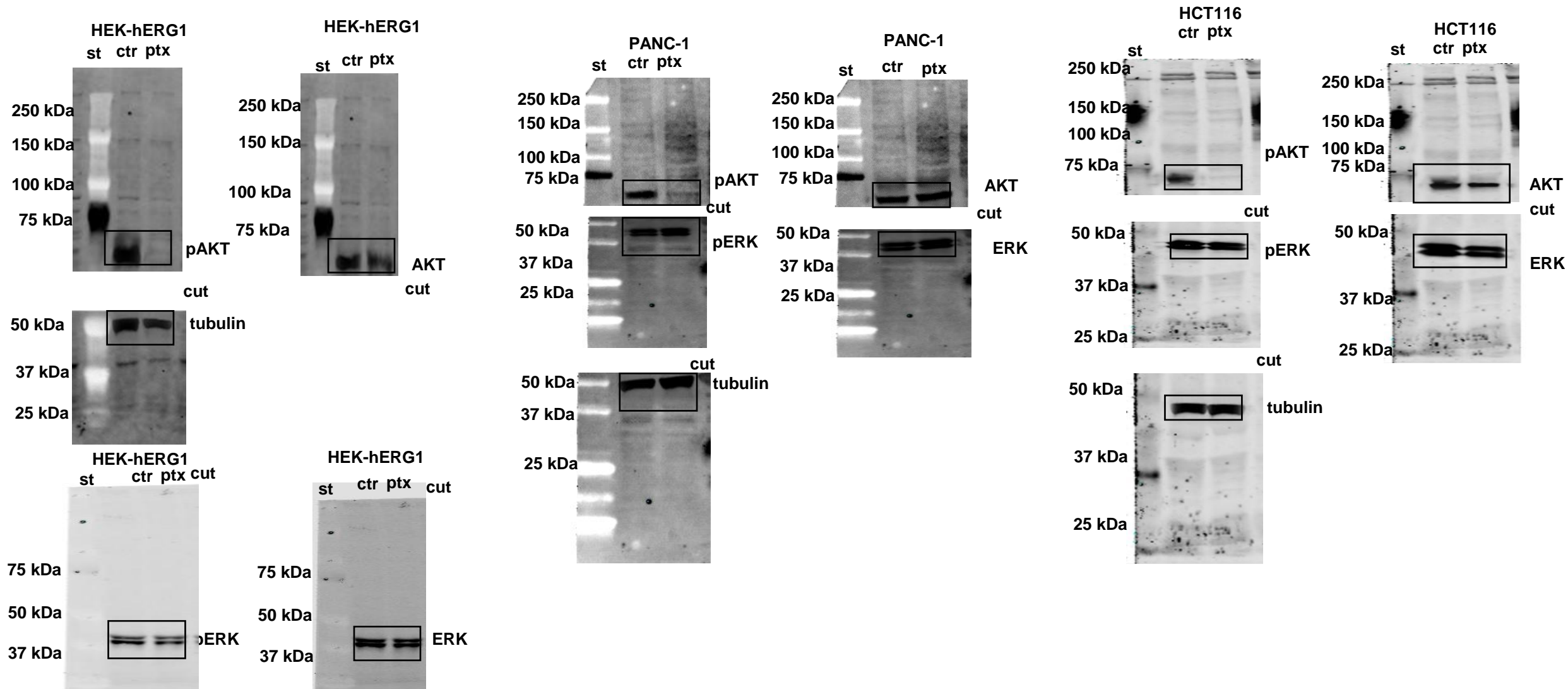

SourceDataForSupplementaryFigureS9B

Supplement: Supplementary file 27 [file LSA-2023-02135_SdataFS9.2.pdf]
