# Supplementary material for: Integrins regulate hERG1 dynamics by girdin-dependent Gαi3: signaling and modeling in cancer cells
Source: Life Sci Alliance. 2023 Nov 3;7(1):e202302135. doi: 10.26508/lsa.202302135 (PMC10624597; doi:10.26508/lsa.202302135)
Supplement: Supplemental Data 1. — Whole-cell network model of hERG1 synthesis, cytoplasmic trafficking, and complex formation with integrins on the plasma membrane. [file LSA-2023-02135_Supplemental_Data_1.pdf]

Whole-cell network model of hERG1 synthesis, cytoplasmic trafficking and complex formation with integrins on the plasma membrane

Contents

1 Quantitative estimation of experimental readouts 1
1.1 Estimation of the total hERG1 expression by quantitative interpretation of IF data 1
1.2 Estimation of the number of hERG1 channels available to activation by electrical stimulation . . . 2
1.3 Estimation of the number of hERG1/β1 integrin complexes on the plasma membrane 2
1.4 Estimation of the glycosylated hERG1/total hERG1 ratio . . . 2
1.5 Estimation of hERG1/β1 integrin complex, immunofluorescence and FACS . . . 2

2 The network model 3
2.1 Numerical solution of the rate equations and fitting procedure . . . 4

1 Quantitative estimation of experimental readouts

1.1 Estimation of the total hERG1 expression by quantitative interpretation of IF data

The use of a digital photon-counting detector for IF-microscopy allowed us to obtain an order-of-magnitude estimation of the expression level of hERG1. IF was performed using direct labeling of the primary antibody with an Alexa 546 (A546) at a degree-of-labeling, DOL = 1.032. The single A546 photon-emission rate for given excitation power, P = 47.5 uW, when located in the focal area, aPSF = 0.223 um^2, and excited at λ = 561 nm is given by

$$R = \frac{P}{a_{PSF}} \frac{\lambda}{hc} \sigma(\lambda) \cdot QY = 7.9 \times 10^6 \text{ photons/s} \tag{1}$$

with the absorption cross-section of A546 at the excitation wavelength, $\sigma(546) = 1.73 \times 10^{-16}$ cm^2, the quantum yield of A546, $QY = 0.79$, the speed of light c, and Planck's constant h. The linear dependency on the excitation power assumed holds only for rates R much smaller than inverse of the excited state lifetime for A546 which is $1/(4.1 \text{ ns}) = 2 \times 10^8$/s. For higher powers saturation has to be taken into account (T. Schmidt, G. J. Schütz, W. Baumgartner H. J. Gruber and H. Schindler (1995) Characterization of Photophysics and Mobility of Single Molecules in a Fluid Lipid Membranes., J. Phys. Chem., 99, 17662). Together with the known detection efficiency of the instrument, $\eta = 0.146$, the pixel dwell time of the microscope, $t_{dw} = 862$ ns, and the number of summed scans, $n = 3$, a single A546 molecule is predicted to yield a signal of

$$S = R\eta t_{dw} = 2.97 \text{ cnts} \tag{2}$$

in our setting used. The ratio of the mean detected signal in the images, $\langle signal \rangle$, to the predicted single molecule signal, S, permits to estimate the density, ρ, of hERG1 channels in the membrane

$$\rho = \frac{\langle signal \rangle}{DOL \cdot m \cdot S} = 1 - 3 \times 10^3 \text{ hERG1/pxl} \quad (3)$$

when considering the proper degree-of-labeling DOL, and the multimeric state of hERG1, $m = 4$. For the determination of the total expression, N, the density is multiplied by the apparent cell area A from the images taken, and considering the oversampling, $o = (2\sigma_{PSF}/pxl)^2 = 8.3$

$$N = \rho \frac{2\langle area \rangle}{o} = 0.5 - 2 \times 10^6 \text{ hERG1/cell} \quad (4)$$

1.2 Estimation of the number of hERG1 channels available to activation by electrical stimulation

An estimate of the number of channels that could be activated by electrical stimulation was obtained by determining the average number of open channels on maximal activation, by whole-cell patch-clamp methods. We thus measured the peak hERG1 tail current -120 mV (which quickly removes inactivation) after fully activating/inactivating the channels by holding the membrane at 0 mV for 15 seconds. We then estimated N by applying equation (1) in the main text, with $Po = 0.8$, and a single channel conductance $\gamma = 2$ pS, as previously reported in experimental conditions comparable to ours' (Kiehn et al., 1996, doi:10.1161/01.cir.94.10.2572; Sanguinetti, C. 2010, doi:10.1007/s00424-009-0758-8). Such estimation gives a number of channels available to activation in the order of 20000, at 90 min after fibronectin engagement.

1.3 Estimation of the number of hERG1/β1 integrin complexes on the plasma membrane

For hERG1/ β1 integrin complex quantification, the signal for the co-immunoprecipitated protein (hERG1) was divided by the signal of the protein used for immunoprecipitation (β1 integrin) and then normalized to the signal of the corresponding protein in the total lysate (β1 integrin input) (Becchetti et al., 2017). The resulting values are indicated as “hERG1/β1 integrin complex”.

1.4 Estimation of the glycosylated hERG1/total hERG1 ratio

The hERG1 signal arising from WBs was quantified by densitometric analysis. The signal obtained from the upper hERG1 bands (weighting roughly 155 kDa and representing the fully glycosylated hERG1 protein) was divided by the signal deriving from the total lysates.

1.5 Estimation of hERG1/β1 integrin complex, immunofluorescence and FACS

For the estimation of these parameters, we have detailed the procedures in the Materials and Methods and figure legends of the main text. Figure 8 of the main text and Table 2 of the present Supplementary file report the corresponding estimated values at the different time points after cell adhesion to FN.

**Symbol**	**Species**
F	Fibronectin
N	hERG1 mRNA
C_c	hERG1 in the ER (non-glycosilated)
C_g	hERG1 in the Golgi (glycosilated)
C_e	hERG1 in endocytotic vesicles (glycosilated)
C	closed-form free hERG1 on the plasma membrane (glycosilated)
C_o	open-form free hERG1 on the plasma membrane (glycosilated)
B	inactive (free) integrin on the plasma membrane
B^*	active integrin on the plasma membrane (fibronectin-integrin complex)
C_B	inactive integrin-hERG1 complex on the plasma membrane (glycosilated)
C_B^*	active integrin-hERG1 complex on the plasma membrane (glycosilated)
A	inactive form of the (catalyst) species that transduces the signal from integrin activation
A^*	active form of the (catalyst) species that transduces the signal from integrin activation
I	inactive form of the (catalyst) species that inhibits the signal from integrin activation
I^*	active form of the (catalyst) species that inhibits the signal from integrin activation
E	inactive form of the internalisation-boosting species
E^*	active form of the internalisation-boosting species

Table 1: List of species in the hERG1 translocation boost network.

2 The network model

The biochemical network that underlies the transduction of integrin-activated transcription, translation and translocation boost of hERG1 channels is depicted in Fig. 1. The species involved are detailed in table 1. There are five conservation laws, namely

$$F + B^* + C_B^* = F_0 \qquad \text{Conservation of fibronectin} \tag{5}$$

$$\beta + C_B + C_B^* = B_0 \qquad \text{Conservation of integrin} \tag{6}$$

$$A + A^* = A_0 \qquad \text{Conservation of activator} \tag{7}$$

$$I + I^* = I_0 \qquad \text{Conservation of inhibitor} \tag{8}$$

$$E + E^* = E_0 \qquad \text{Conservation of internalizer} \tag{9}$$

where $\beta = B + B^*$ is the total number of free integrins not in complex with a channel. The rate equations read

$$\frac{dN}{dt} = k_n + k_{n+}(B^* + C_B^*) - k_{-n}N \tag{10}$$

$$\frac{dC_c}{dt} = (k_c + k_{c+}A^*)N - (k_g + k_{g+}A^*)C_c \tag{11}$$

$$\frac{dC_g}{dt} = (k_g + k_{g+}A^*)C_c - (k_{tg} + k_{tg+}A^*)C_g \tag{12}$$

$$\frac{dC_e}{dt} = k_eE^*C - (k_{te} + k_{de})C_e \tag{13}$$

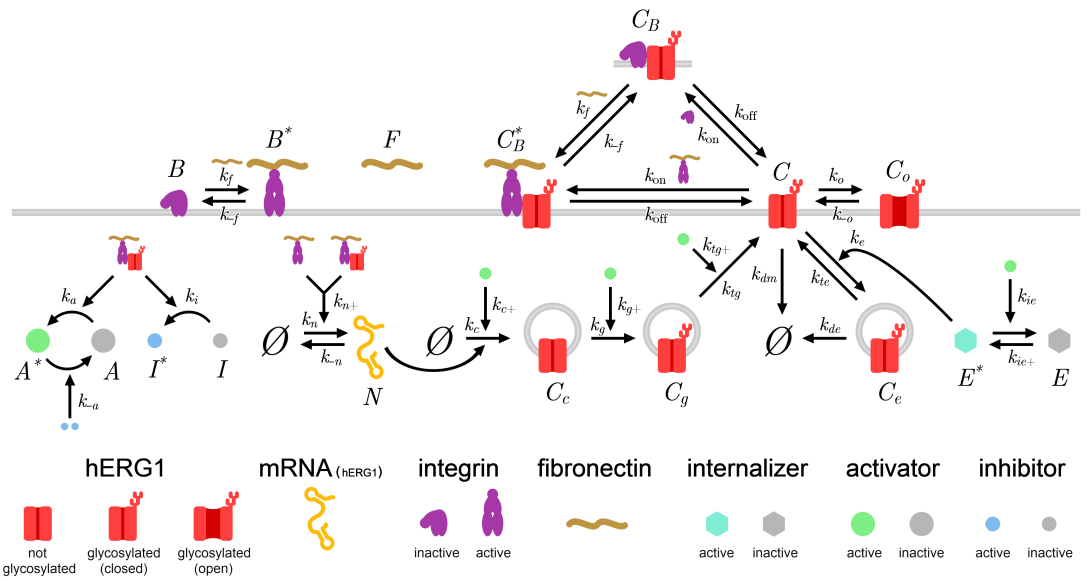

Figure 1: Scheme of the whole-cell reaction network underlying integrin-activated translocation boost of hERG1.

$$\begin{aligned}
\frac{dC}{dt} &= (k_{tg} + k_{tg+}A^*)C_g + k_{te}C_e + k_{\text{off}}(B_0 - \beta) + k_{o-}C_o + \\
&\quad -(k_{\text{on}}\beta + k_o + k_{dm} + k_e E^*)C \qquad (14) \\
\frac{dC_o}{dt} &= k_o C - k_{o-}C_o \qquad (15) \\
\frac{dC_B^*}{dt} &= k_{\text{on}}B^*C + k_f(B_0 - C_B^* - \beta) - (k_{\text{off}} + k_{-f})C_B^* \qquad (16) \\
\frac{dB^*}{dt} &= k_f\beta + k_{\text{off}}C_B^* - (k_{-f} + k_f + k_{\text{on}}C)B^* \qquad (17) \\
\frac{d\beta}{dt} &= k_{\text{off}}(B_0 - \beta) - k_{\text{on}}\beta C \qquad (18) \\
\frac{dA^*}{dt} &= k_a(A_0 - A^*)C_B^* - k_{a-}(I^*)^2 A^* \qquad (19) \\
\frac{dI^*}{dt} &= k_i(I_0 - I^*)C_B^* \qquad (20) \\
\frac{dE^*}{dt} &= k_{ie+}(E_0 - E^*) - k_{ie}A^*E^* \qquad (21)
\end{aligned}$$

where $k_f = k_f^0(F_0 - B^* - C_B^*)$.

2.1 Numerical solution of the rate equations and fitting procedure

The fitting procedure has been implemented in custom Python codes developed independently by the authors. The fits were performed by minimising a cost function consisting of the squared differences between the numerical solution of the rate equations and the experimental measurements summed over the time points set in the experiments and the experimental read-outs

Parameter	HEK	PANC−1	HCT−116	Units
k_c	0.402	1.43	1.5	s^{-1}
k_g	0.0524	0.139	0.205	s^{-1}
k_{tg}	0.00304	0.00206	0.00154	s^{-1}
k_e	3.55×10^{-5}	0.000227	0.000352	(# molecules/cell)$^{-1}$ s^{-1}
k_{te}	0.0608	0.0218	0.0292	s^{-1}
k_{de}	0.105	0.18	0.253	s^{-1}
k_{off}	107	346	234	s^{-1}
k_{on}	4.24×10^{-5}	0.000113	8.82×10^{-5}	(# molecules/cell)$^{-1}$ s^{-1}
k_{-f}	8.05×10^{-6}	8.37×10^{-5}	8.79×10^{-5}	s^{-1}
k_a	4.91×10^{-8}	1.18×10^{-7}	1.92×10^{-7}	(# molecules/cell)$^{-1}$ s^{-1}
k_{a-}	0.000258	0.000873	0.000486	(# molecules/cell)$^{-2}$ s^{-1}
k_i	1.23×10^{-9}	1.17×10^{-9}	1.73×10^{-9}	(# molecules/cell)$^{-1}$ s^{-1}
k_{ie}	65.2	79.2	71.8	(# molecules/cell)$^{-1}$ s^{-1}
k_n	163	100	86.4	(# molecules/cell) s^{-1}
k_{n+}	0.000776	0.000898	0.00109	s^{-1}
k_{-n}	0.0134	0.01	0.0116	s^{-1}
k_{c+}	0.00804	0.0286	0.03	(# molecules/cell)$^{-1}$ s^{-1}
k_{g+}	0.000698	0.00185	0.00274	(# molecules/cell)$^{-1}$ s^{-1}
k_{tg+}	0.000108	7.32×10^{-5}	5.46×10^{-5}	(# molecules/cell)$^{-1}$ s^{-1}
k_{dm}	0.105	0.18	0.253	s^{-1}
k_f^0	1.75×10^{-5}	6.29×10^{-6}	8.42×10^{-6}	(# molecules/cell)$^{-1}$ s^{-1}
k_{ie+}	0.132	0.225	0.317	s^{-1}
k_{o-}	8	8	8	s^{-1}
k_o	0.15	0.15	0.15	s^{-1}
B_0	1.709×10^7	1.709×10^7	1.709×10^7	(# molecules)
A_0	1.5×10^3	1.5×10^3	1.5×10^3	(# molecules)
I_0	1.5×10^3	1.5×10^3	1.5×10^3	(# molecules)
E_0	10^3	10^3	10^3	(# molecules)
F_0	2.4×10^5	2.4×10^5	2.4×10^5	(# molecules)

Table 2: Values of the the best-fit values of the floating parameters of the model. The last seven bottom lines report parameters that have been kept at a fixed value during the fits.

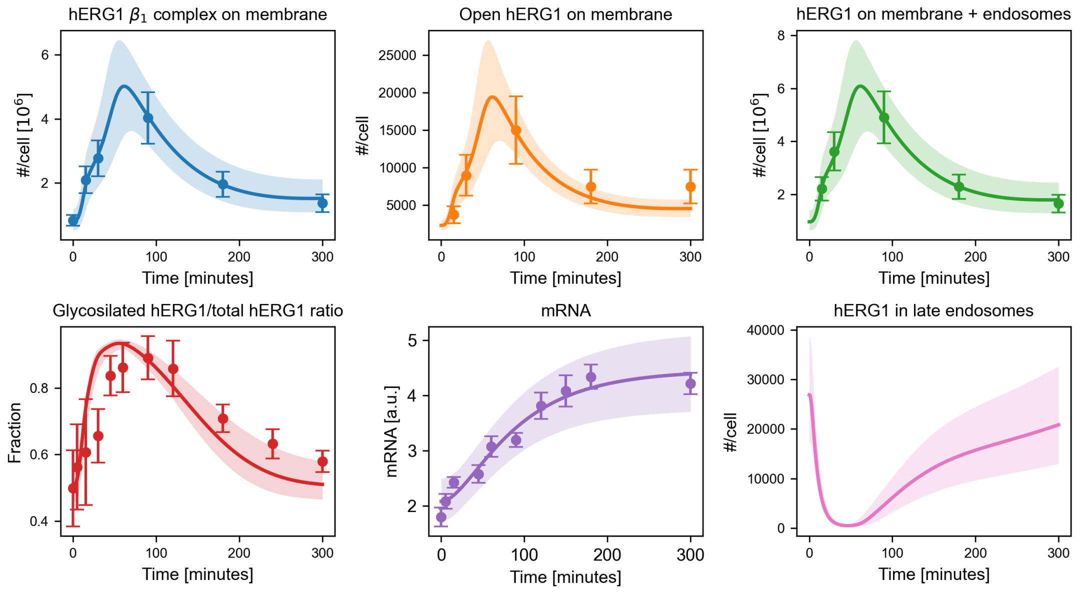

Figure 2: **Expression, translocation and complex formation of hERG-1 upon adhesion of HEK cells on fibronectin**. Comparison of the experimental data (symbols) with the best-fit solution of the rate equations (10) (solid lines). The shaded areas mark the observed variability regions corresponding to 100 different independent sets of the floating parameters obtained by randomly perturbing each of them within ± 10 % of its best-fit value.

selected to the included in the optimisation procedure, namely (i) hERG1 β_1 complexes on the membrane, open hERG1 on the membrane, total hERG1 on the membrane + endosomes, glycosilated hERG1/total hERG1 ratio and total mRNA copy number.

The minimisation routine consisted in a Monte Carlo minimisation whereby two free parameters were selected randomly at each iteration and increased or reduced (with probability 1/2) by a fixed factor, with the new values accepted only if the new choice led to a reduction of the cost function. We found that this simple routine worked extremely effectively with specific choices of the inflation/deflation multiplicative factor (the same for all parameters) and of the allowed variability ranges for the free parameters (typically rather wide ones).

At each call of the cost function, the whole set of rate equations was solved numerically with the current values of the parameters. The rate equations were solved via the `odeint` function included in the Python `scipy` package (which in turn uses the FORTRAN library `odepack`).

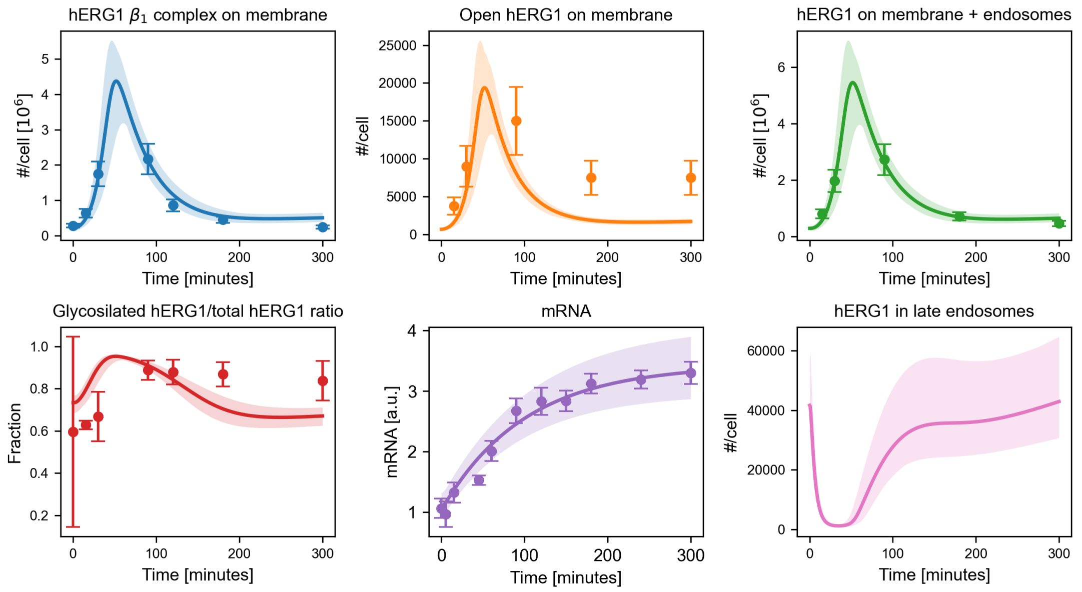

Figure 3: **Expression, translocation and complex formation of hERG-1 upon adhesion of PANC-1 cells on fibronectin**. Comparison of the experimental data (symbols) with the best-fit solution of the rate equations (10) (solid lines). The shaded areas mark the observed variability regions corresponding to 100 different independent sets of the floating parameters obtained by randomly perturbing each of them within ± 10 % of its best-fit value. The experimental points in the top central panel are those from HEK cells (see Fig. 2). These were not included in the cost function used to fit simultaneously the experimental readouts, but are shown here for comparison. The solid line in the same panel should thus be interpreted as the time course of the number of open hERG1 channels on the PANC-1 membrane predicted by our model.

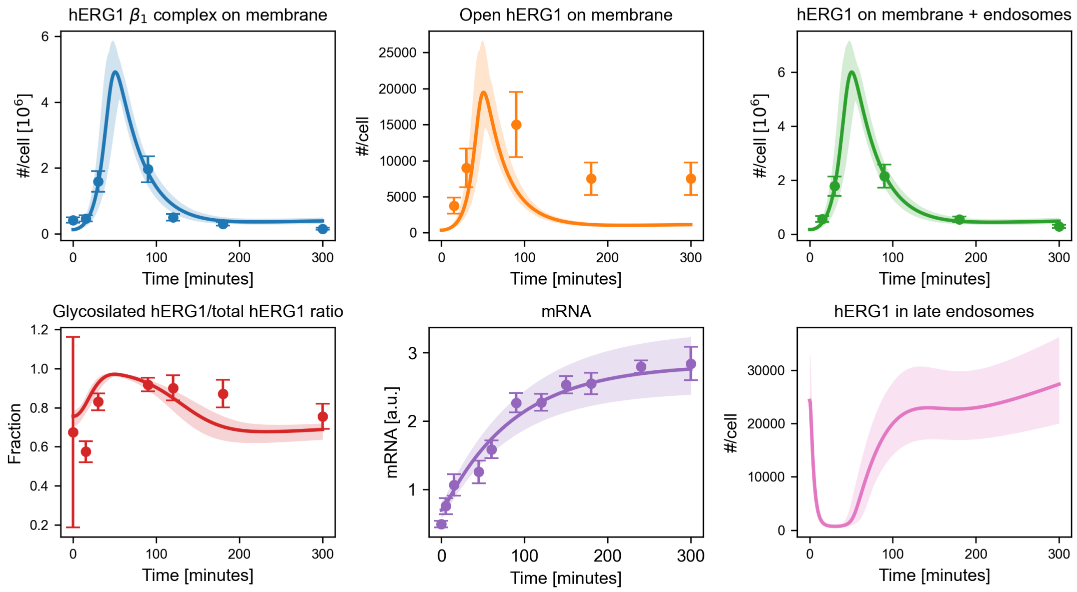

Figure 4: **Expression, translocation and complex formation of hERG-1 upon adhesion of HCT-116 cells on fibronectin**. Comparison of the experimental data (symbols) with the best-fit solution of the rate equations (10) (solid lines). The shaded areas mark the observed variability regions corresponding to 100 different independent sets of the floating parameters obtained by randomly perturbing each of them within ± 10 % of its best-fit value. The experimental points in the top central panel are those from HEK cells (see Fig. 2). These were not included in the cost function used to fit simultaneously the experimental readouts, but are shown here for comparison. The solid line in the same panel should thus be interpreted as the time course of the number of open hERG1 channels on the HCT-116 membrane predicted by our model.
