# Supplementary material for: Integrins regulate hERG1 dynamics by girdin-dependent Gαi3: signaling and modeling in cancer cells
Source: Life Sci Alliance. 2023 Nov 3;7(1):e202302135. doi: 10.26508/lsa.202302135 (PMC10624597; doi:10.26508/lsa.202302135)
Supplement: Supplementary file 13 [file LSA-2023-02135_SdataFS5.1.pdf]

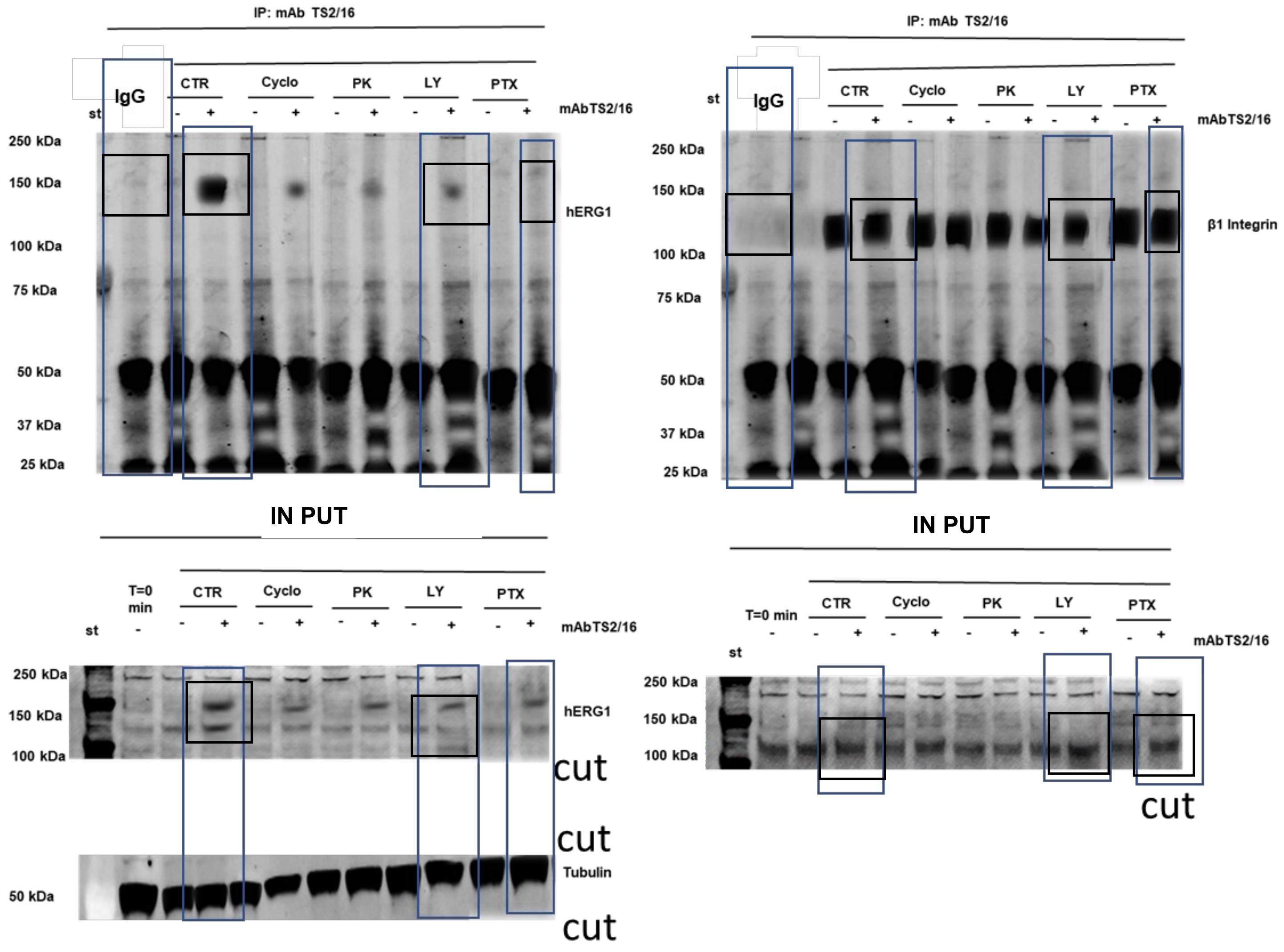

SourceDataForSpplementaryFigureS5A
